# Predicting target-ligand interactions using protein ligand-binding site and ligand substructures

**DOI:** 10.1186/1752-0509-9-S1-S2

**Published:** 2015-01-21

**Authors:** Caihua Wang, Juan Liu, Fei Luo, Zixing Deng, Qian-Nan Hu

**Affiliations:** 1 School of Computer, Wuhan University, 430072 Wuhan, PR China; 2 Key Laboratory of Combinatorial Biosynthesis and Drug Discovery (Ministry of Education) and Department of Pharmaceutical Sciences, 430071 Wuhan, PR China

## Abstract

**Background:**

Cell proliferation, differentiation, Gene expression, metabolism, immunization and signal transduction require the participation of ligands and targets. It is a great challenge to identify rules governing molecular recognition between chemical topological substructures of ligands and the binding sites of the targets.

**Methods:**

We suppose that the ligand-target interactions are determined by ligand substructures as well as the physical-chemical properties of the binding sites. Therefore, we propose a fragment interaction model (FIM) to describe the interactions between ligands and targets, with the purpose of facilitating the chemical interpretation of ligand-target binding. First we extract target-ligand complexes from sc-PDB database, based on which, we get the target binding sites and the ligands. Then we represent each binding site as a fragment vector based on a target fragment dictionary that is composed of 199 clusters (denoted as fragements in this work) obtained by clustering 4200 trimers according to their physical-chemical properties. And then, we represent each ligand as a substructure vector based on a dictionary containing 747 substructures. Finally, we build the FIM by generating the interaction matrix M (representing the fragment interaction network), and the FIM can later be used for predicting unknown ligand-target interactions as well as providing the binding details of the interactions.

**Results:**

The five-fold cross validation results show that the proposed model can get higher AUC score (92%) than three prevalence algorithms CS-PD (80%), BLM-NII (85%) and RF (85%), demonstrating the remarkable predictive ability of FIM. We also show that the ligand binding sites (local information) overweight the sequence similarities (global information) in ligand-target binding, and introducing too much global information would be harmful to the predictive ability. Moreover, The derived fragment interaction network can provide the chemical insights on the interactions.

**Conclusions:**

The target and ligand bindings are local events, and the local information dominate the binding ability. Though integrating of the global information can promote the predictive ability, the role is very limited. The fragment interaction network is helpful for understanding the mechanism of the ligand-target interaction.

## Background

Through various high-throughput experimental projects for analyzing the genome, transcriptome and proteome, we are beginning to understand the genomic spaces. Simultaneously, the high-throughput screening of large-scale chemical compound libraries with various biological assays enable us to explore the chemical space. However, our knowledge about the relationship between the chemical and genomic spaces is very limited. For example, the PubChem database at NCBI [1] stores information on millions of chemical compounds, but the number of compounds with information on their target protein is very limited [2]. Therefore, there is a strong incentive to develop new methods capable of detecting these potential target-ligand interactions efficiently.

Due to time and cost limitations of experimental approaches, a number of predictive approaches attempt to predict target-ligand relationships in silico. The traditional computational predictive methods roughly fall into two categories: target-based approaches and ligand-based approaches [3]. Target-based approaches mainly utilize the target information to predict. Molecular docking is a target-based approach [4,5], which predicts the preferred orientation by conformation searching and energy minimization. Docking could provide excellent conformation, but it is difficult to find a rank/evaluation function to select which orientation is more appropriate [6]. Another target-based method is comparing target similarities, which compares the targets of a given ligand by sequences, EC number, domains, 3D structures, etc. Ligand-based methods compare candidate ligands with the known ligands of a given target to make a prediction [3]. Three-dimensional quantitative structure-activity relationship (3D-QSAR) is a typical ligand-based model [7], which indirectly reflect non-bonding interaction characteristics between the ligand and target. The most widely used 3D-QSAR methods are comparative molecular field analysis (CoMFA) and comparative molecular similarity (CoMSIA). CoMFA first aligns the ligands capable of binding to a given target, and then measure field intensities around the aligned ligands by different atom probes (force field-based). Finally, the measured field intensities are regressed with the active values and the regression equation is applied to predict interactions. Moreover, we can map the coefficients of CoMFA back into 3D space to obtain a 3D-QSAR model, which could guide the optimization of lead compounds [7].

Recently, some methods considering both the target and ligand information have been proven to be promising in drug design and discovery. Jacob *et al*. applied the EC Number (Enzyme Commission number) and PubChem fingerprints (a set of molecular substructures) [8] to represent targets and ligands respectively, and proposed a pairwise support vector machine (pSVM) method to predict target-ligand interactions [9]. Laarhoven *et al*. described the targets and ligands by sequences and compound 3D structures respectively, and introduced the target-ligand interaction network to build the prediction model [10]. Bleakley *et al*. proposed Bipartite Local Model (BLM), which integrated the ligand-based and target-based methods to generate a comprehensive prediction [11]. BLM has been further studied by Xia *et al*., Laarhoven *et al*. and Mei *et al*.[12,10,13]. The BLM shows a very good predictive ability, however, it cannot deal with the situation that both the ligand and target are unseen in the training set. Yamanishi *et al*. represented the genome space with sequences and target profiles, and the chemical space with compound 3D structures and ligand profiles, and then generated a uniform "pharmacological space" to build the prediction model [2]. Cheng *et al*. applied the mass distribution property from physics on the target-ligand network to predict the target-ligand interactions [14]. Cao *et al*. integrated the genome and chemical space into random forests to obtain a better predictive ability [15].

**Figure 1:**
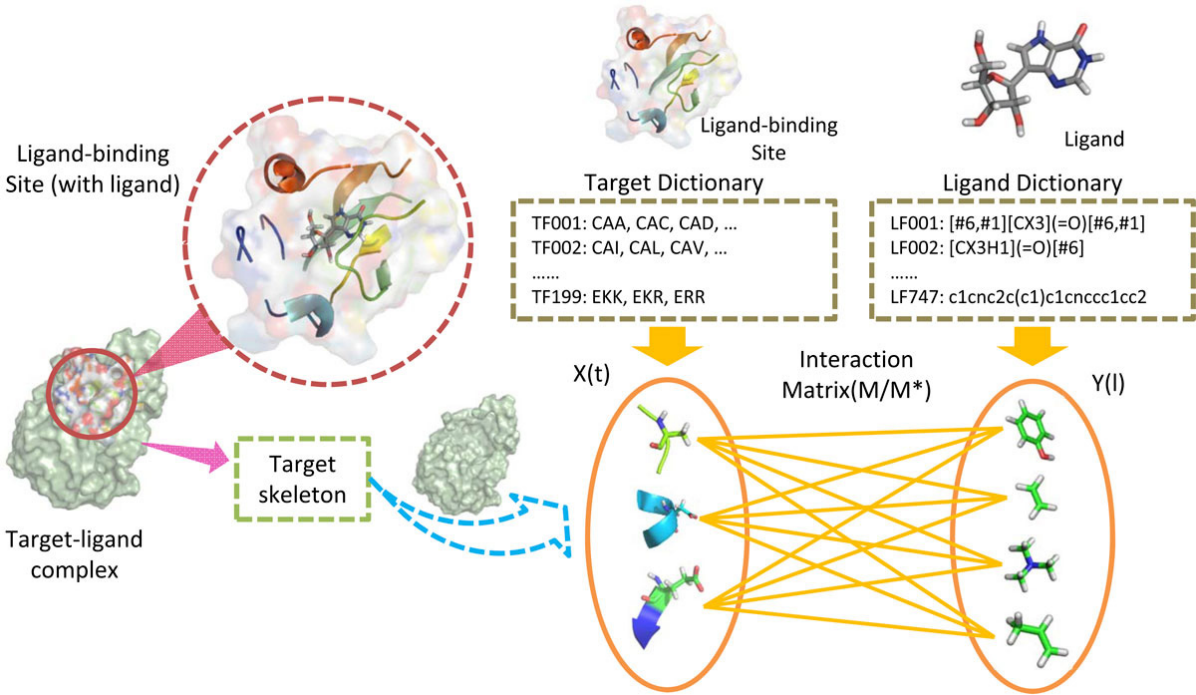
**Fragment interaction model (FIM)**. Each element in the target dictionary is a trimer cluster, and trimers belonging to the same cluster share similar chemical properties; Each element in the ligand dictionary is a chemical substructure. The binding sites of the targets are represented by fragments (trimer clusters) information and the ligands are encoded with substructures (fragments) information. We assume that the (binding site) fragment-(ligand) fragment interactions facilitate the site-ligand binding. The interaction between a binding site fragment and a ligand substructure is determined by their distance.

However, most of the existed methods consider the target as a whole, resulting that it is difficult to investigate the latent binding mechanism between target and ligand. In other words, we know little about how the chemical space interacts with the genomic space. In this article, based on the fact that the target-ligand interaction is more of a local event, we use the binding sites (local information) instead of the whole target to describe the genomic space. Furthermore, we assume that the fragment-fragment interactions determine the target-ligand interactions. Thus we break the binding sites and ligands into fragments, and propose fragment interaction model (FIM) to figure out a clean picture of how the chemical space interacts with the genomic space (Figure 1).

## Materials and methods

### Data set

**Figure 2:**
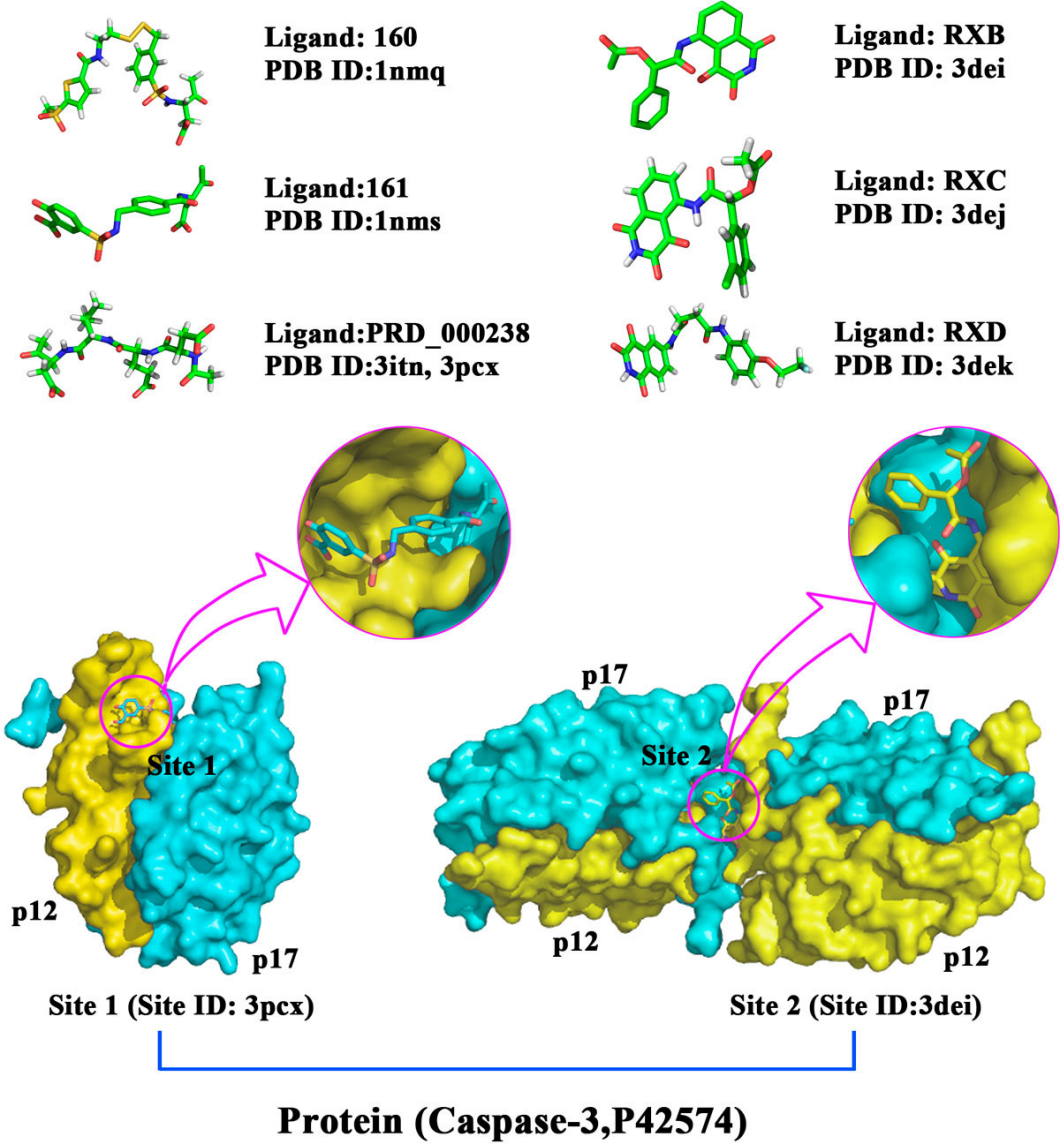
**Illustration of the target-ligand binding**. A target might possess more than one binding sites, and a binding site might bind with many ligands. The data set is organized in three levels: targets, sites and ligands.

We extracted all human targets and ligands from sc-PDB database [16], which is an annotated archive of the druggable binding sites extracted from the Protein Data Bank [17]. In this work, we set the amino acid residues possessing at least one atom within eight angstroms around the ligand as binding sites. A target might possess more than one binding sites, and each binding site might interact with several ligands (Figure 2). After removing the redundance and checking the consistency, we got 836 targets and 2710 corresponding ligands. Among them, 782 single binding site targets interact with 1988 ligands to form 2561 interaction pairs, and other multiple binding sites targets interact with 722 ligands to form 854 interaction pairs. According to a published method [9], for each binding site, we generated as many negative site-ligand pairs as the known positive pairs by combining the site with randomly chosen ligands among the other sites' ligands (excluding those known to interact with the given target). Of course, this protocol may generate false negative data for some ligands could actually interact with the site whereas they have not been experimentally tested. Totally, 6830 site-ligand pairs include in our data set (Table 1).

**Table 1:** Statistics of the data set

	Target	Ligand	Pairs
One-site	782	1988	5122
Multi-site	54	722	1708
Total	836	2710	6830

### Representation of targets' binding sites based on target dictionary

In this work, we made use of the target dictionary provided by Nagamine and Sakakibara [18] to represent the biding sites. The dictionary is organized as three layers: the amino acids, trimers and trimer clusters. In the first layer, each amino acid is first represented by 237 items of physical-chemical properties [19,18], such as residue volume, polarizability and solvation free energy. And then, a principal component analysis (PCA [20]) is applied to reduce the dimension. As a result, each amino acid is described by a 5-dimensional feature vector. In the second layer, twenty amino acids are permutated and combined into 4200 trimers. Each trimer ***α***_*tri*_(*α*_01_, *α*_11_, *α*_12_) is mapped into a 5-dimensional vector space [18] as follows:

(1) $$\alpha_{tri}\left(\alpha_{01},\alpha_{11},\alpha_{12}\right)=\alpha \left(\alpha_{01}\right)+\frac{\alpha \left(\alpha_{\text{11}}\right)+\alpha \left(\alpha_{\text{12}}\right)}{4}$$

where ***α***(*α*_01_), ***α***(*α*_11_), ***α***(*α*_12_) and ***α***_*tri*_(*α*_01_, *α*_11_, *α*_12_) are the 5-dimensional vectors. *α*_01 _is the center (major) amino acid, *α*_11 _and *α*_12 _are the left and right amino acid (subordinate) respectively. There is no location difference between *α*_11 _and *α*_12_, that means ***α***_*tri*_(*α*_01_, *α*_11_, *α*_12_) and ***α***_*tri*_(*α*_01_, *α*_12_, *α*_11_) are equivalence. In the third layer, the hierarchical clustering (Ward's algorithm [21]) is used to cluster 4200 trimers into 199 clusters [19,18]. All clusters constitute the dictionary.

Therefore, we first broke the binding site sequences into trimers. For example, the amino acid sequence NGMGN produces three trimers G(NM), M(GG) and G(MN). Since G(NM) and G(MN) are equivalence, we could combine them by adding a count. Then, we casted all the trimers into 199 clusters, and counted the occurring frequency of each cluster in every binding site. Finally, all cluster frequencies were normalized to unit *L*_2 _norm to obtain the feature vectors with 199 dimensions for the binding sites. For example, the sequence NGMGN can be represented as following:

(2) $$\begin{array}{c} Bs\left(NGMGN\right)\\ \begin{array}{cccccc} & \ldots & c\left(G\left(NN\right),G\left(MN\right),\ldots \right) & \ldots & c\left(M\left(GG\right),M\left(AG\right),\ldots \right) & \ldots \\ = & & & & & \\ & (\ldots & \frac{2}{\sqrt{5}} & \ldots & \frac{1}{\sqrt{5}} & \ldots ) \end{array} \end{array}$$

where *B_s_*(·) denotes a binding site feature vector. *c*(·) denotes a cluster, for example, *c*(*G*(*N N*), *G*(*M N*),...) represent a cluster that contains *G*(*N N*), *G*(*M N*), etc. Because the trimers in the same cluster own similar chemical properties, the clusters can be viewed as chemical "groups", based on which the ligand binding sites are decomposed into fragments.

### Generation of ligand dictionary and ligand representation

Representation of chemical space involves two steps, defining a dictionary and de-scribing ligands as features. We have integrated sever data sources to make the dictionary (data shows in supplementary materials). In PubChem database, there are 881 predefined chemical substructures. We made some modification on the fin-gerprints to gear with our model. First, the single atoms and bonds were removed because they are not in the same structural level with trimers. Second, some sub-structures, such as benzene were removed; because they are too common to serve as a discriminately feature. Third, functional groups/fingerprints of molecular in Check-mol were integrated [22]. Finally, we generated a dictionary with 747 substructures. Based on the dictionary, each ligand was represented by a 747-dimensional binary vector whose element indicates the presence or absence of each substructure by 1 or 0.

### Construction of fragment interaction model

As described above, all binding sites could be described as a matrix ***S ***= (***s***_**1**_, ***s***_**2**_,⋯,***s***_***m***_)*^T^*, where *m *is the number of binding sites. ***s_i _***= (*s*_*i*1_, *s*_*i*2_,⋯,*s*_*ip*_)*^T ^*denotes the *i*-th binding site, where *s*_*ik *_corresponds the *k*-th fragment of the *i*-th binding site and *p *is the number of site features/fragments. Meanwhile, all ligands could be described as a matrix ***L ***= (***l***_**1**_, ***l***_**2**_,⋯,***l***_***n***_)*^T^*, where *n *is the number of ligands. ***l***_***j ***_= (*l*_*j*1_, *l*_*j*2_,⋯,*l*_*jq*_)^*T *^denotes the *j*-th ligand, where *l*_*jk *_indicates the presence or absence of ligand the *k*-th substructures of the *j*-th ligand and *q *is the number of ligand features. The interaction data set is denoted as *D *= {(*i*_1_, *j*_1_), (*i*_2_, *j*_2_),⋯,(*i_c_*, *j_c_*)}, where *i_k _*= 1, 2,⋯,*m*; *j_k _*= 1, 2,⋯,*n*; (*i*_*k*_, *j*_*k*_) is the *k*-th site-ligand pair and *c *is the number of site-ligand interaction pairs. $s_{i_{k}}$ and $l_{j_{k}}$ are the site and ligand vectors in the *k*-th interaction pair respectively. ***y ***= (*y*_1_, *y*_2_,⋯,*y_c_*) denotes the labels of interaction pairs, where *y*_*k *_∈ {1, −1} indicate the positive and negative interaction respectively. Because fragments of targets and ligands are in a physically interaction distance (within 8 angstrom), it is reasonable to assume that there exist inherent chemical interactions between target and ligand features, and the sum of feature interactions determinates the target-ligand interaction. Therefore, the proposed approach is called fragment interaction model (FIM, Figure 1) and it can be expressed as the following equation:

(3) $$F\left(s_{*},l_{*}\right)=s_{*}^{T}{Ml}_{*}$$

where ***s*_∗ _**represents a binding site and ***l*_∗ _**represents a ligand. ***s*_∗ _**and ***l*_∗ _**might be unseen to the data set. ***M ***represents genomic and chemical spaces interaction matrix/network. If *sign*(*F *(***s*_∗_**, ***l*_∗_**)) is 1, we predicted a positive interaction, otherwise we predicted a negative interaction (*sign*(·) is the sign function, return −1 and 1).

It is easy to solve parameters ***M ***in Equation 3 by logistic regression. However, it is inconvenient to expand to include more information. Therefore, mathematics transformation is conducted, and Equation 3 changes into Equation 4.

(4) $$F\left(s_{*},l_{*}\right)=w^{T}\left(s_{*}⊗l_{*}\right)$$

where ***w ***is a vector vision of features interaction matrix ***M***, and ⊗ denotes tensor product. Obviously, Equation 3 and Equation 4 are equivalent. For convenience, we denoted:

(5) $$\psi \left(s_{*},l_{*}\right)=s_{*}⊗l_{*}$$

It is easy to fit ***w ***through support vector machine (SVM). Based on the Lagrange dual theory, Equation 4 can be rewritten as its dual form on the data set.

(6) $$F\left(s_{*},l_{*}\right)=\overset{c}{\underset{k=1}{\sum }}\alpha_{k}y_{k}\psi {\left(s_{i_{k}},l_{j_{k}}\right)}^{T}\psi \left(s_{*},l_{*}\right)$$

Where *α_k _*is dual variable and *y_k _*is the label of the interaction pair (*i_k_*, *j_k_*) Equation 6 demonstrates that for a given site-ligand pair (***s*_∗_**, ***l*_∗_**), it only relates with inner product of the support site-ligand pairs (*α_k _*≠ 0). Therefore, we should only care 0 about the inner product of support site-ligand pairs and the site-ligand pair to predict.

(7) $$\psi {\left(s_{*1},l_{*1}\right)}^{T}\psi \left(s_{*2},l_{*2}\right)={\left(s_{*1},⊗l_{*1}\right)}^{T}\left(s_{*2}⊗l_{*2}\right)=\left(s_{*1}^{T}s_{*2}\right)\left(l_{*1}^{T}l_{*2}\right)$$

where (***s*_∗1_**, ***l*_∗1_**) and (***s*_∗2_**, ***l*_∗2_**) are two pairs of site-ligands. Equation 7 is very important because it transforms tensor product into inner product. On one hand, we avoided calculating the tensor product, which always means large computing load. One the other hand, inner product facilitates us to design kernels and add more information by kernel trick. For convenience, we denoted:

(8) $$K_{loc}\left(s_{*1},s_{*2}\right)=s_{*1}^{T}s_{*2},\;K_{lig}\left(l_{*1},l_{*2}\right)=l_{*1}^{T}l_{*2}$$

Then Equation 7 can be changed as:

(9) $$K_{loc-lig}\left(s_{*1},l_{*1};s_{*2},l_{*2}\right)=K_{loc}\left(s_{*1},s_{*2}\right)K_{lig}\left(l_{*1},l_{*2}\right)$$

Because we utilized linear kernel, therefore, the above Equations are invertible and the genomic and chemical interaction matrix ***M***, on the data set, can be described as:

(10) $$M=\overset{c}{\underset{k=1}{\sum }}\alpha_{k}y_{k}s_{i}l_{j}^{T}$$

Although Equation 7 have been mentioned in many papers [9,11,23], the kernels in those works were nonlinear and irreversible (because of kernel trick), thus we known little about how the genomic space interact with chemical space. In this paper, we adopted linear kernel without kernel trick, so that the genomic and chemical interaction matrix could be calculate through Equation 10, which rendered the model to be chemical interpretable.

### Representative methods for comparison

In order to evaluate the proposed method in this paper, we chosen three representative methods for comparison: chemical substructures and protein domains correlation model (CS-PD) [23], bipartite local model with neighbor-based interaction-profile inferring (BLM-NII) [11] and random forest (RF) [15].

• CS-PD: Proteins were described by domains and ligands were represented by substructures in CS-PD model. Sparse canonical correspondence analysis (SCCA) algorithm was applied to recognize the physical-chemical factors between the domains and substructures. In prediction phase, the domain and substructure physical-chemical factors in a given target-ligand pair were added to generate a discriminant value. If the value was higher than a threshold, the target and ligand were predicted to interact with each other.

• BLM-NII: On one hand, excluding target *t_i_*, make a list of all other known targets of ligand *l_j_, *as well as a separate list of the targets not known to be targeted ligand *l_j. _*The known targets were given a label +1 and the others a label −1. Then, look for a classification rule that tried to discriminate the +1-labeled data from the −1-labeled data using the available genomic sequence data for the targets. This rule was applied to predict the label of target *t_i _*and ligand *l_j. _*On the other hand, fixing the same target *t_i _*and excluding ligand *l_j_*, make a list of all other known ligands targeting *t_i_*, as well as a list of ligands not known to target *t_i_*. Similar with before, ligands known to target *t_i _*were given the label +1 and the others were given the label −1. We looked for a classification rule that tried to discriminate the +1-labeled data from the −1-labeled data, using the available chemical structure data for the ligands. This rule was also used to predict the label of target *t_i _*and ligand *l_j. _*At last, the two results were combined to generate a final label. For new targets or ligands, a neighbor-based interaction-profile inferring was applied to get an interaction profile.

• RF: The targets were described as CTD (Composition-Transition-Distribution, [15]) features. The ligands were represented as fingerprints. Then, the two kinds of features were combined into a vector to generate data set. Finally, random forest (RF) was employed to predict interactions.

## Results

### Investigation on the interaction data

**Figure 3:**
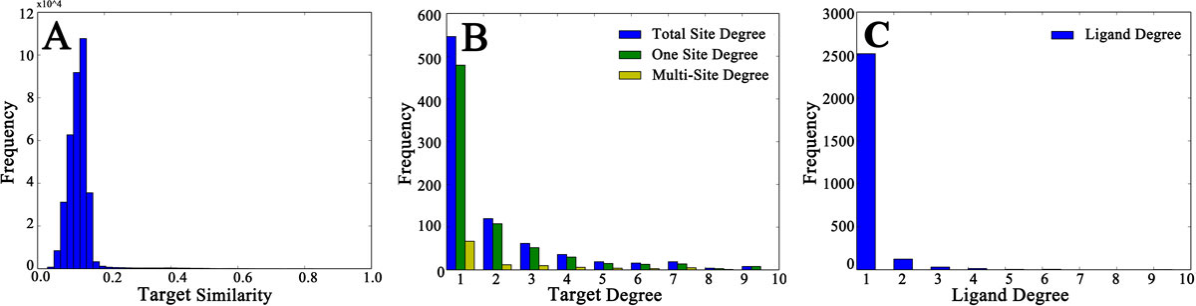
**Investigation of the data set**. A) The distribution of target sequence similarities B) The degree distributions of targets C) The degree distribution of the ligands

We have investigated the collected data set in this work. We measured the similarity of the selected targets with Smith-Waterman score (Figure 3A), and found that the similarities of vast majority of targets are low (<0.2), indicating that the homology of the selected targets in the data set is weak. X-ray and other biology studies suggest that a number of proteins contain more than one ligand-binding sites. For example, some enzymes possess two or more binding sites, one for substrate and another for activator/inhibitor. Therefore, we constructed a sites-ligand interaction network using a bipartite graph to check the degree distributions of both binding sites and ligands (Figure 3B and 3C). From Figure 3B we can see that each of the most binding sites bind with only one ligand, which is consistent with the fact that the binding of target and ligand is specific. Figure 3C shows that more than 95% ligands interact with only one site. In all, we can infer that the targets in the data set are low in homology, the connections of site-ligand bipartite graph are sparse and the average degree of binding sites is larger than that of ligands.

### Comparison results

Since the original representative methods were implemented with different data sets, it is unfair to directly compare them with our method. Therefore, we implemented the algorithms on our data set, and evaluated the performances of all methods with multiple criteria, such as accuracy (ACC, the percentage of correct predictions), precision (the percentage of true positive instances in all predicted positive predictions), recall (the percentage of predicted true positive predictions in all true positive instances) and area under receiver operating characteristic curve (AUC, comprehensive evaluation of classifier performance, between 0.5 to 1, the larger the better). The result is shown in Table 2.

**Table 2:** Comparison result of the prediction performances

	ACC	Precision	Recall	AUC
FIM	0.835	0.848	0.821	0.916
CS-PD	0.565	0.552	0.562	0.799
BLM-NII	0.727	0.712	0.812	0.858
RF	0.743	0.756	0.719	0.851

Table 2 shows that the ACC and AUC scores of CS-PD are 56.5% and 79.9% respectively, which means the correct prediction rate is only slightly higher than random guess (the expect correct rate of random guess is 50%) and the comprehensive performance is not good. We guess that the poor performance of CS-PD is due to lacking of powerful classifier and it only serves as a feature extraction approach. BLM-NII preforms good in our data set, but not as well as in its origin data set (Yamanishi's "Gold Standard"). The AUC score of BLM-NII is 85.8% in our data set, while it is more than 98% in all four categories (enzyme, ion channel, GPCR, nuclear receptor) in its origin data set. The difference of data set could be the main cause of the AUC difference. It is a pity that not all the crystal structures of the targets in Yamanishi's data set are determined, and we could not perform our approach in the "Gold Standard". The ACC and AUC scores of RF are 0.743% and 0.851% respectively, which are similar with BLM-NII. The bagging ensemble procedure might promote the prediction ability of RF model. The ACC and AUC of FIM are 82.7% and 91.6% respectively, which is much higher than that of CS-PD, BLM-NII and RF. The ACC and AUC score is promoted more than 10% and 5% respectively, compared with state-of-the-art (BLM-NII). In short, the FIM have shown remarkable predictive ability and outperforms other three approaches in our data set.

### The role of global information in the binding

Because the intensity of intermolecular interaction decreases rapidly with the increasing distance, we can infer that local information may dominate the binding procedure. However, the local binding sites are inevitably influenced by the other part of target. We adopt the target sequence, obtained from the KEGG database [24], similarity score as global information. The sequence similarities are measured in nor-malized Smith-Waterman scores [25]. $K_{glo}\left(t,t^{\prime }\right)=SW\left(t,t^{\prime }\right)/\sqrt{SW\left(t,t\right),SW\left(t^{\prime },t^{\prime }\right)}$ where *t *and *t' *are protein sequences, *SW *(·, ·) is the original Smith-Waterman s-core, and *K_glo _*is a target global similarity matrix (global information). Finally, the global and local information are integrated by kernel trick, as follows:

(11) $$K_{tar}\left(s_{*1},s_{*2}\right)=\lambda K_{glo}\left(t_{*1},t_{*2}\right)+\left(1-\lambda \right)K_{loc}\left(s_{*1},s_{*2}\right)$$

where ***s*_∗1 _**and ***s*_∗2 _**are binding sites, ***t*_∗1 _**and ***t*_∗2 _**are target sequences corresponding to ***s*_∗1 _**and ***s*_∗2_**, and *λ *is the ratio of global information. After the introduction of global information, the kernels are no longer linear. We attempted to estimate the role of global information in the binding procedure by increasing the ratio *λ*. With the increase of global information (*λ*), the AUC score first increases, until *λ *= 0.3, then, the score reaches the maximum and further increasing of global information (*λ*) would result in the AUC score decreasing (Table 3). Although AUC score varies with *λ*, it only varies in a narrow range (from 0.916 to 0.922), which implies that the global information only has a limited influence on prediction accuracy.

**Table 3:** Local-global trade-off

*λ*	0.0	0.1	0.2	0.3	0.4	0.5
AUC	0.916	0.919	0.920	0.922	0.919	0.918

**Figure 4:**
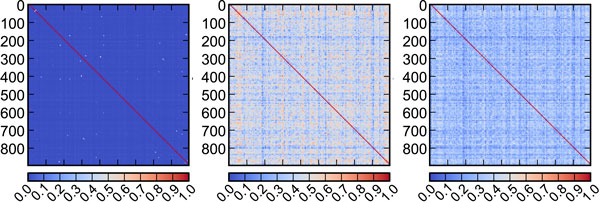
**Kernel matrix of global and local information**. The left penal is the global kernel matrix; the middle penal is the local kernel matrix; the right penal is the global-local kernel matrix (*λ *= 0.3). The global-local kernel matrix and the local kernel matrix are similar, because the norm of global kernel matrix is small with regard to local kernel matrix.

Another approach to analyze the importance of global information is to measure the difference of the target kernel matrix (including global and local information, *λ *= 0.3) and the local kernel matrix (Figure 4). The left, middle and right panels of Figure 4 are the global, global-local (*λ *= 0.3) and local kernel matrices respectively. Figure 4 shows that a large area of the global kernel matrix is blue, which means that most values in the global kernel matrix are small. Comparing to global kernel matrix, the values in the local kernel matrix are much larger and the local kernel matrix determine the global-local kernel matrix. That would be the reason why the weight of global information is as high as 30%, while AUC score varies less than 1% (Table 3).

Based on the above facts, it is reasonable to infer that the fluctuation caused by global information is limited and local information dominates the binding predictive accuracy, which support our assumption that target-ligand binding is a local event.

### Fragment interaction network analysis

In this section, we first give a brief overview of fragment interaction matrix. Then we investigate the underlying chemical mechanisms of fragments interactions.

**Figure 5:**
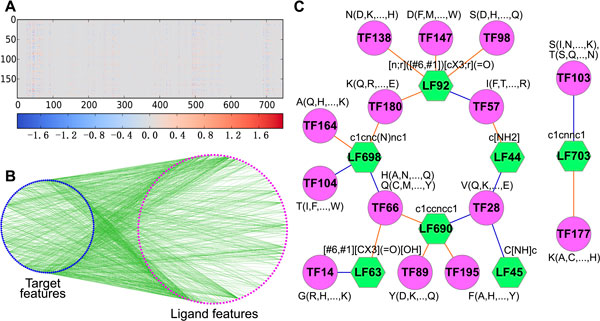
**Interaction network analysis**. A) An overview of feature interaction network. The horizontal ordinate and longitudinal coordinates are ligand features and target features respectively. B) The import interaction network (a subnetwork of fragment interaction network). C) The top twenty interactions. The interactions can reflect the chemical interaction.

An obvious feature of the fragment interaction matrix (Figure 5A) is that the values can be positive and negative, which means some fragment interactions are in favor of binding, and others not. Another obvious feature is that most of the values are close to zeros, which means the connections between site and ligand fragments are sparse. The sparse connection implies a site fragment only could recognize a small number of ligand fragments, which could reflect the specificity during the target-ligand binding procedure. Although there are 148653 (199 *∗ *747) elements in the matrix, only those whose value is larger than 0.1 are viewed as significant (the average standard error is 0.1). As a result, there are 9243 significant interactions in the network. During the significant interactions, the interaction values larger than 0.25 (top 20%) are regarded as import. Figure 5B shows the import fragment interactions.

According to the hypothesis, the feature interactions reflect the chemical interaction, as a result, it is necessary to investigate whether the feature interactions response the hypothesis. Since the number of interactions is large, we only analyze the top twenty interactions (Figure 5C), the others could be analyzed similarly. In Figure 5C, the first letter of site fragment is the center amino acid of the trimer cluster, and the letters in the parenthesis represent the subordinate amino acids. The smarts (a kind of molecular patterns) represent ligand fragments. The Figure 5C suggest that the feature interactions reflect the chemical interaction well, which in consistent with the hypothesis. For example, the major amino acid of site fragment 147 (TF147) is Aspartic (short for D), which could interact with ligand fragment 92 (LF92, containing keto group) through hydrogen bond, if the distance and orientation are appropriate. In some situations, the major amino acid of a target feature could not form significant interaction with ligand feature, but the subsidiary amino acid could. For example, the major amino acid of site fragment 57 (TF57) is isoleucine (short for I), which is a hydrophobic amino acid. Isoleucine could not interact with ligand fragment 44 (LF44), which contains amino group. However, the subsidiary amino acid of site fragment 57, such as threonine (short for T) and arginine (short for R) can form hydrogen bond with ligand fragment 44, if the distance and orientation are appropriate.

## Discussion and conclusion

In this work, we consider binding is a local event and emphasize the local information in target-ligand interaction prediction. We apply site-ligand interactions instead of target-ligand interactions and propose a chemical interpretable model to cover the site-ligand interactions. We first extract the ligand-binding sites from target-ligand complexes. Then we break the binding sites and ligands into fragments so that they can be encoded as fragment vectors based on target and ligand dictionary respectively. Finally, we assume that the fragments interactions determine the site-ligand interaction and propose a model, fragment interaction model (FIM), to generalize the assumption. The proposed model demonstrates higher AUC score (92%) with respect to two prevalence algorithms CS-PD (80%), BLM-NII (85%) and RF (85%). In addition, the fragment interaction network origined from FIM is chemical interpretable. Comparing to BLM-NII, RF and CS-PD model, it require crystal structure to extract local information (binding site) in FIM, which hinder the applying of FIM sometimes. However, with the increasing determination of protein crystal structures and the developing molecular modeling technique, we can model a 3D structure by computer, and extract the binding site.

Compared with traditional target-based or ligand-based approaches, the proposed FIM method has the advantages of finding target candidates and ligand candidates simultaneously. Moreover, FIM can predict the interaction between previously unseen targets and ligand candidates. Different with other target-ligand based methods, our method emphasizes the basic chemical interactions between amine acids and ligand fragments, which is more general and could be applied beyond drugtarget interactions. Furthermore, we no longer represent the target as a whole but extract the ligand-binding sites from target-ligand complexes and apply the binding sites to describe the genomic space. For one hand, representing the genomic space by binding sites allows us provide site-ligand interaction prediction, which is important for multi-site targets. For another hand, the binding sites are local, which facilitate to achieve chemical interpretable model. Along this way, we break the binding sites and ligands into fragments, and regard the fragment interactions as genomic and chemical space interactions. We know clearly about how the genomic space interacts with chemical space under FIM.

In all, we highlight the local information during the binding process and attempt to figure out a clear relationship between the genomic and chemical spaces. The proposed model (FIM) applies the ligand binding sites as local information and views the binding site and ligand fragment interactions as genomic and chemical space interactions. The fragment interactions are straightforward and chemical interpretable, and the fragment interaction network reflect the chemical interactions. The comparison result shows that FIM outperforms other three approaches. The investigation on the role of global information shows that the local information dominate the predictive accuracy and integrating of the global information might promote the predictive ability to a very limited extent.

## Competing interests

The authors declare that they have no competing interests.

## Authors' contributions

CHW, JL and QNH developed the methodology. CHW executed the experiments, CHW and JL wrote this paper. CHW, JL, FL, ZXD and QNH revised the manuscript. All authors read and approved the final manuscript.
